# Occlusal management for a patient with aural symptoms of unknown etiology: a case report

**DOI:** 10.1186/1752-1947-1-85

**Published:** 2007-09-12

**Authors:** Kengo Torii, Ichiro Chiwata

**Affiliations:** 1 Torii dental clinic, 1-23-2 Ando, Aoi-ku, Shizuoka-shi, 420-0882, Japan; 2 Chiwata dental clinic, 2-1-3 Gofuku-cho, Aoi-ku, Shizuoka-shi, 420-0031, Japan

## Abstract

**Background:**

Although the discrepancy between the habitual occlusal position (HOP) and the flat bite plate-induced occlusal position (BPOP) (regarded as the muscular physiological reference position) has been recently reported to be related to symptoms of temporomandibular disorders (TMDs), it still remains unclear whether the occlusal equilibration in the reference position is effective to resolve TMD-related discrepancy and symptoms. Aural symptoms (otalgia, tinnitus, vertigo et cetera) have been included under TMD symptoms.

**Methods:**

To examine the effect of occlusal equilibration for the treatment of TMDs, occlusal equilibration was performed for a patient with aural symptoms (otalgia, tinnitus and vertigo) of unknown etiology in the right ear. An occlusal analysis was performed on this patient with dental models mounted on an articulator after relieving painful symptoms by an appliance therapy and a discrepancy was identified (p < 0.005). Occlusal equilibration in the BPOP was then performed for the patient by selective tooth grinding, because it was estimated that the interocclusal space between upper and lower occlusal surfaces would be rectified by selective grinding.

**Results:**

At completion of treatment, the discrepancy was not significant (p > 0.25), and the patient's right condyle had shifted 2.8 mm posteromedially in the horizontal plane, and the left condyle had shifted 1.0 mm laterally in the voluntarily closed position from the previous HOP. The aural symptoms of the patient were resolved, and there has been no recurrence to date after a two-year follow-up period.

**Conclusion:**

An occlusal analysis should be performed in patients exhibiting TMD symptoms to identify the presence or absence of any discrepancy between the HOP and the BPOP. If a discrepancy exists, occlusal equilibration should be attempted in the reference position.

## Background

Aural symptoms have been reported to sometimes occur in temporomandibular disorders (TMDs) [1,2], and there have been many reports of improvement of aural symptoms after various treatments for TMD [3,4]. A variety of hypotheses have been proposed to explain the aural symptoms [5,6]. The relation between TMD and occlusion has been the subject of considerable debate and controversial. However, it has been recently demonstrated that a discrepancy between the habitual occlusal position (HOP) and the flat bite plate-induced occlusal position (BPOP) is associated with TMD symptoms [7]. The HOP is obtained by voluntary jaw closing while a patient is sitting in the upright position. The BPOP is obtained during voluntary jaw closing while the patient is sitting in the upright position and after wearing an anterior flat bite plate that covers the 6 upper anterior teeth and both first premolars for a brief time, and then removing it. The BPOP has been regarded as the physiologic masticatory muscular position and a reference position for occlusal analysis. The effect of occlusal equilibration in BPOP on TMD symptoms has also been inferred [7,8]. Therefore, it is worth to examine the occlusal equilibration in the BPOP for TMD patient. The following case report describes a patient who presented with aural symptoms of unknown etiology. Occlusal equilibration in the BPOP was found to be effective against aural symptoms, and it might be effective against other TMD symptoms.

## Case presentation

**Figure 1:**
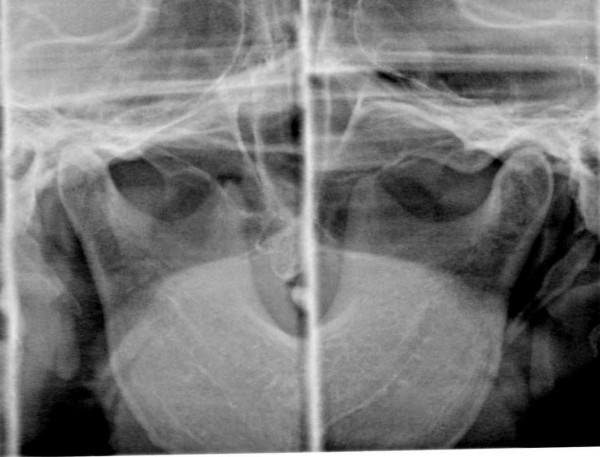
Tomographyic image showing an unclear right TMJ in the HOP before treatment.

A 73-year old woman presented with the chief complaint of severe pain and tinnitus in the right ear. She reported that when performing karaoke 2 years previously, she suddenly felt a strong impact on her right ear and developed such severe rotatory vertigo that she was unable to remain standing and had to crawl on the floor. She was diagnosed as having Ménière's disease by two independent otorhinolarygologists and was treated with isosorbide, mecobalamin and ibudilast. However, when the drugs failed to improve her severe symptoms, she came to our dental clinic. The patient's medical history was unremarkable. No impairment of mouth opening, no deviation of the opening path, and no noises in either of the temporomandibular joints (TMJs) was detected. The patient reported pain on palpation of the right TMJ and tenderness of the right lateral pterygoid muscle. Twenty-nine teeth present, and the upper right third molar was slightly extruded because there was no opposing tooth. Occlusion was anatomically normal. The right TMJ appeared unclear on the TMJ computed tomography (CT) images in the HOP from the first consultation (Fig. 1). The cause of the lack of clarity was unknown, but it seemed to be attributable to inflammation, or to the eccentricity the condyle in the glenoid fossa.

For this patient, three HOP records were obtained by voluntary jaw closing at the first visit, while the patient was seated in an upright position with the occlusal plane parallel to the floor using a vinyl polysiloxane bite registration material (GC, Tokyo, Japan). Since an anterior flat bite plate has been recommended as a provisional appliance to decrease painful symptoms [4], an anterior flat bite plate was directly fabricated in the patient's mouth using clear self-curing acrylic resin (GC, Tokyo, Japan), which the patient wore every night for a week. At the second visit, (one week after the first visit), her otalgia had ceased, and the intensity of the tinnitus was greatly diminished. The BPOP records were obtained during voluntary jaw closing, in the upright position and after wearing the bite plate for five minutes using the same material as in the HOP. Three BPOP records were obtained. To examine the difference between the HOP and BPOP, three-dimensional measurements were performed on the modified articulator using previous records [7]. The difference between the HOP and BPOP was significant using an analysis of variance (ANOVA) for a two factor experiment with repeated measurements for both position factors (p < 0.005). An occlusal analysis was performed on dental models mounted on an articulator (Gnatho-Tech, Shizuoka, Japan) with a BPOP wax (Kerr, Romulus, MI, U.S.A.) record. One of several therapies (selective tooth grinding, prosthodontic and orthodontic treatments and orthognathic surgery) may be selected for occlusal equilibration in the BPOP, according to the degree of discrepancy between the HOP and BPOP. In the present case, extraction of the right upper third molar was selected first, because of premature occlusal contact in the BPOP. Subsequently, tooth grinding was selected, because it was estimated that the interocclusal space between the upper and lower occlusal surfaces would be rectified by selective grinding. The instability of the patients' occlusion in BPOP and the need of occlusal equilibration were explained to the patient using the articulated dental models on an articulator. She gave oral informed consent for performing occlusal equilibration in the BPOP. The upper right third molar was extracted. After verifying the consistency of the BPOP using a split cast method on 3 consecutive visits at 3-day intervals on the articulator, selective grinding in the BPOP was performed during 5 appointments over 3 weeks until occlusal contacts of the premolar and molar teeth were achieved on both sides. For selective grinding in the patient's mouth, the anterior bite plate was worn in the moth and the patient was asked to tap and slide her anterior teeth against the plate for five minutes while in an upright position. The plate was removed, and the patient was asked to close her jaw until tooth contact was made and then hold that position. Premature contact was located in the mouth by marking and pulling with occlusal tape (Hanel, Langenau, Germany). The consistency of the BPOP was confirmed at every selective grinding time.

**Figure 2:**
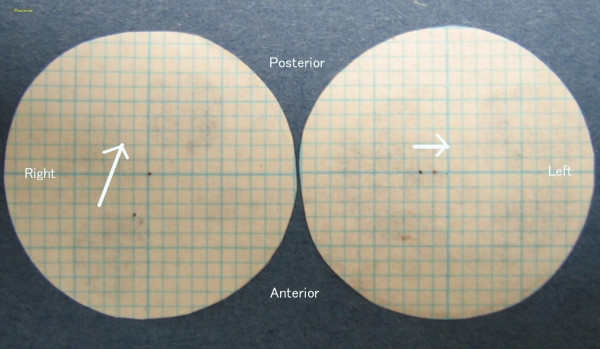
The shifts recorded on both condylar areas of the articulator. The degree of the shift indicates the actual change in the patient's condyles because the intercondylar distance of the articulator is adjusted to the same distance as that of the patient.

**Figure 3:**
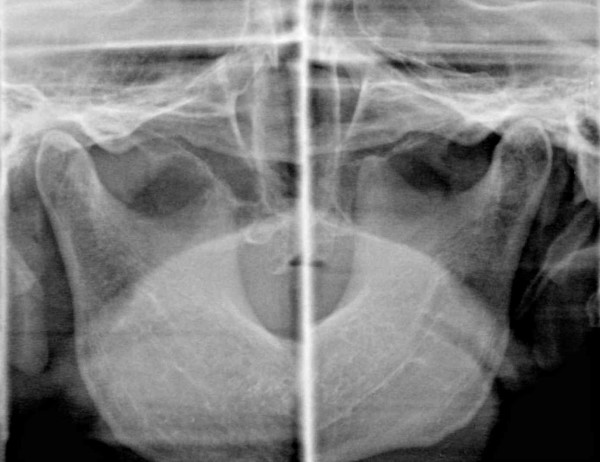
Tomographic image showing a clear right TMJ in the HOP after treatment.

At the completion of the selective tooth grinding, the difference between the HOP and BPOP was not significant (p > 0.25), and the right condyle in the voluntarily closed position had shifted 2.8 mm posteromedially in the horizontal plane, and the left condyle had shifted 1.0 mm laterally from the previously recorded HOP at the first visit (Fig. 2), and the right TMJ on tomography images appeared to be clear than that obtained at the first examination (Fig. 3). The patient's aural symptoms resolved, and there has been no recurrence in symptoms and no reappearance of the discrepancy between the HOP and BPOP to date after a two-year follow-up period.

## Discussion

There have been many reports of relief or complete elimination of otalgia, tinnitus, vertigo, and deafness by various treatments for TMDs [3,4]. However, the outcomes of these various conservative therapies for TMDs in relation to the aural symptoms have always been an improvement in symptoms rather than a cure, because they were not established on the basis of the pathogenic mechanism underlying the development of the aural symptoms. The occlusal equilibration in our patient was performed based on evidence of a significant association between the difference between the HOP and BPOP and TMJ sounds [7]. The large shift in the position of the right condyle in the HOP in our patient after treatment means that the right condyle had deviated anterolaterally before treatment. In regard to the development of aural symptoms, Williamson [5] hypothesized that vertigo was caused by noxious pain stimuli to the peridiskal tissues inducing constriction of the internal auditory and posterior auricular arteries thus decreasing the blood supply. Myers [6] described the disk displacement in the TMJ as being accompanied by stretching of the posterior laminae and stretching or tearing of the lateral collateral ligament. Myers claimed that this injury caused chronic inflammation with extension of the inflammatory areas. This occurs especially in the carotid sheath area, and causes adhesion of arteries, veins, and nerves of the sheath, such that the indirect tension along the carotid sheath caused by the adhesion interferes with the pressure-regulating function of the saccus endolymphaticus. The end result of this process is increased endolymphatic pressure and development of aural symptoms. In our patient, it was considered that the pathological conditions described by Williamson [5] or Myers [6] probably occurred with large deviations of the right condyle. Moyers [8] described that the persistence of occlusal disharmony in centric relation might cause a new reflex pattern of pathways to be repeatedly used, causing the new position of the mandible to represent the intercuspal position. The discrepancy between the HOP and BPOP in our patient might have emerged as a result of persistent premature occlusal contact of the upper right third molar during its extrusion.

A proper occlusal analysis should be performed with the BPOP record obtained after relief of the painful symptoms by appliance therapy. Selective tooth grinding in voluntary jaw closing has been considered unreliable; however, the reproducibility of the jaw closing can be improved by wearing a bite plate. When performing selective grinding in the BPOP, it is important to check to see whether the BPOP is consistent, because the BPOP in TMD patients is variable [7]. The consistency of BPOP wax records on three consecutive visits at 3- to 7-day intervals needs to be confirmed on an articulator. In the present study, the selective tooth grinding in the BPOP seemed to provide a "cure" for our patient who had vertigo, tinnitus and otalgia.

## Conclusion

An occlusal analysis should be carried out in patients with TMD-related symptoms to identify whether a discrepancy exists between the HOP and BPOP (the latter being regarded as a physiological reference position). If a discrepancy does in fact exist, the occlusal equilibration in the reference position should be attempted.

## Competing interests

The author(s) declare that they have no competing interests.

## Authors' contributions

KT conceived of the study, and participated in the designing the study and perfomed the occlusal analysis and selective grinding. IC carried out the three-dimensional measurements and performed the statistical analysis.

The authors read and approved the final manuscript.
